# Taurine Activates BMP-2/Wnt3a-Mediated Osteoblast Differentiation and Mineralization via Akt and MAPK Signaling

**Published:** 2019-11

**Authors:** Minsu PARK, Hyeon Kyeong CHOI, Jeung Hee AN

**Affiliations:** 1. Department of Biomedical Chemistry, Konkuk University, Chungju, Korea; 2. Department of Food and Nutrition, KC University, Seoul, Korea; 3. Department of Food Science and Technology, Seoul National University of Science & Technology, Seoul, Korea

**Keywords:** Taurine, Osteoporosis, Osteoblast, Ovariectomized rat

## Abstract

**Background:**

We aimed to elucidate the preventive effects of taurine against osteopenia in ovariectomized (OVX) rats and the mechanisms by which taurine regulates osteoblastogenesis in vitro and in vivo.

**Methods:**

The effects of the taurine on human osteoblast MG-63 cell differentiation and osteoblastogenesis effect in OVX rat were examined Konkuk University in 2018 by evaluating osteoblast differentiation, and expression of osteoblast-specific factors by western blotting analysis.

**Results:**

Taurine supplementation significantly improved alkaline phosphatase (ALP) activity and mineralization in a concentration-dependent manner. Further, taurine induced the expression of osteogenic growth factors such as bone morphogenetic protein-2 (BMP-2), runt-related transcription factor 2 (RUNX2), small mothers against decapentaplegic 1/5/8 (SMAD1/5/8), wingless-type MMTV integration site family member 3A (Wnt3a), and collagen type 1 (COL-1) via mitogen-activated protein kinase (MAPK) and serine/threonine protein kinase (Akt). Moreover, the RUNX2 activity of the taurine-treated group was enhanced by protein–protein interactions such as Wnt3a-induced *p*-AKT/RUNX2 and BMP-mediated SMADs/MAPK/RUNX2 interactions.

**Conclusion:**

Our in vitro and in vivo results suggested that taurine can be considered as a potential therapeutic candidate agent for preventing bone loss in postmenopausal osteoporosis.

## Introduction

Osteoporosis is a disease that causes excessive bone loss, especially in menopausal women (1). The bone loss is attributed to decrease in the number of osteoblasts and increase in the number of osteoclasts, which results in the formation of bone pores (2). The major factors governing osteoporosis are smoking, alcohol abuse, menopause, hormone deficiency, aging, and nutritional status. These factors also reduce the bone mineral density (BMD) and increase bone fracture risk (3). In addition, genetic and environmental factors, including dietary pattern, contribute to osteoporosis (4, 5). Among the dietary factors, protein intake is known to have various influences on bone health (6).

Taurine (2-aminoethanesulfonic acid) is a major amino acid detected in the brain, heart, eyes, muscle, and liver of mammals (7, 8). Taurine has various biological actions such as anti-obesity (9) and antioxidant (10) activities, retinal degeneration prevention (11, 12), and breast cancer suppression (13, 14). The consumption of taurine-rich seafood has been shown to be associated with the reduced risk of metabolic diseases such as obesity and hypertension (9, 15). Further, taurine has been shown to protect against DNA damage owing to its antioxidative action (10). Taurine is thought to be found at 0.1% of the body weight in bone cells and promotes bone formation and inhibits bone loss (16–18). It affects the expression of connective tissue growth factor through cell signaling pathways in osteogenic cells (17). Recent studies have shown that taurine increases alkaline phosphatase (ALP) activity and collagen synthesis in osteoblast-like UMR-106 cells (19). A taurine transporter was shown to be expressed in human osteoblast-like MG-63 cells (8). This transporter mediated the activation of ALP activity and osteocalcin secretion (8) and inhibition of osteoclastogenesis (20). However, studies on the osteoblastic mechanisms of taurine are limited (8).

Therefore, we aimed to elucidate the mechanism of action of taurine and its effect on osteoblast differentiation and postmenopausal osteoporosis, using human osteoblast-like cells and ovariectomized (OVX) rats. To our knowledge, this is the first study to elucidate the effect of taurine on osteoblasts in the OVX animal model.

## Materials and Methods

### MG-63 cell culture and reagents

Human osteoblast-like cells (MG-63) was purchased from the Korean Cell Bank (Seoul, Korea) and cultured in Rosewell Park Memorial Institute (RPMI) medium. The media contained 10% fetal bovine serum (FBS) and 1% streptomycin-penicillin (100 U/mL). All cells were incubated at 95% humidity, 5% CO_2_, and 37°C. Taurine (≥99%) was purchased from Sigma-Aldrich (Darmstadt, Munich, Germany).

### ALP activity

The rate of hydrolysis of *p*-nitrophenyl phosphate (*p*-NPP) was measured as an indicator of ALP activity in MG-63 differentiated using 50 μg/mL of ascorbic acid and 5 mM of β-glycerophosphate. In brief, 5 × 10^4^ cells were dispensed into each well of a 96-well plate and cultured for 24 h; subsequently, various concentrations (80, 150, and 300 μg/mL) of taurine were added to RPMI, and the cells were cultured for 72 h. After treatment, 0.1% Triton X-100 and *p*-NPP were added, and the cells were incubated at 37°C for 60 min. Next, the ALP activity was measured at 405 nm by using a microplate reader (Biochrom Asys, Cambridge, UK).

### Bone mineralization analysis by using alizarin red S staining

After 14 days of taurine treatment, the degree of mineralization was analyzed using alizarin red S staining. In brief, all cells were washed twice with phosphate buffered saline (PBS) and fixed with 70% ethanol for 1 h at 4°C. Next, the cells were stained with 40 mM alizarin red S dissolved in deionized water (pH 4.2) for 15 min. After the Alizarin Red S solution was removed, the cells were incubated in PBS for 15 min and rinsed once with fresh PBS. Subsequently, the cells were decolorized with 10% cetylpyridinium chloride in 10 mM sodium phosphate for 15 min. The stained extract was dispensed into 96-well plates, and the absorbance was measured at 550 nm.

### Animals and diet

All in vivo experiments were approved by the Animal Care and Use Committee of Konkuk University (IACUC; Approval Number, KU 18030).

Forty 10-week-old female Wistar rats (body weight, 227 ± 13 g) were obtained from Doo Yeol Biotech (Seoul, Republic of Korea). The animals were reared under humane care in an air-conditioned environment (23 ± 1°C) with a 12 h light/dark cycle (07:00 h on, 19:00 h off) free access to food pellets and water. After 1-week acclimatization, the rats were anesthetized with diethyl ether, and both the ovaries were excised. The rats were randomly divided into four groups. Three groups received ovariectomy, and the sham group received an incision, but no ovariectomy. After the 1-week of adaptation, the sham and positive control groups were fed normal diet (0.6% calcium, TD.97191), and the negative control was fed TD.95027 diet; the taurine group was fed TD.95027 + 1% taurine diet (0.01% calcium, TD.95027 + taurine). In addition, all rats were provided the experimental diet and deionized water *ad libitum* for 12 weeks. All rats were weighed at every 10 days. After the 12 weeks, the rats were anesthetized and killed. The tibia bones of each animal were dissected, sampled, and stored at −20°C. The left tibia was used for RT-PCR and western blotting analysis after micro-CT and breaking force analyses. The right tibia was used to measure the calcium content of the tibia and for histological examination.

### Western blot analysis

Western blot was performed using cultured cells and tibia, and compared using β-actin as an internal control for other proteins (21).

### Statistical analysis

Statistical analysis was performed using SPSS 22 (SPSS Inc., CA, IL, USA). The standard deviation and averages of all variables were calculated, and the differences between the groups were analyzed using one-way analysis of variance and Duncan’s multiple range test. Values with *P* <0.05 were considered statistically significant.

## Results

### Taurine stimulates ALP activity and bone mineralization

**Fig. 1:**
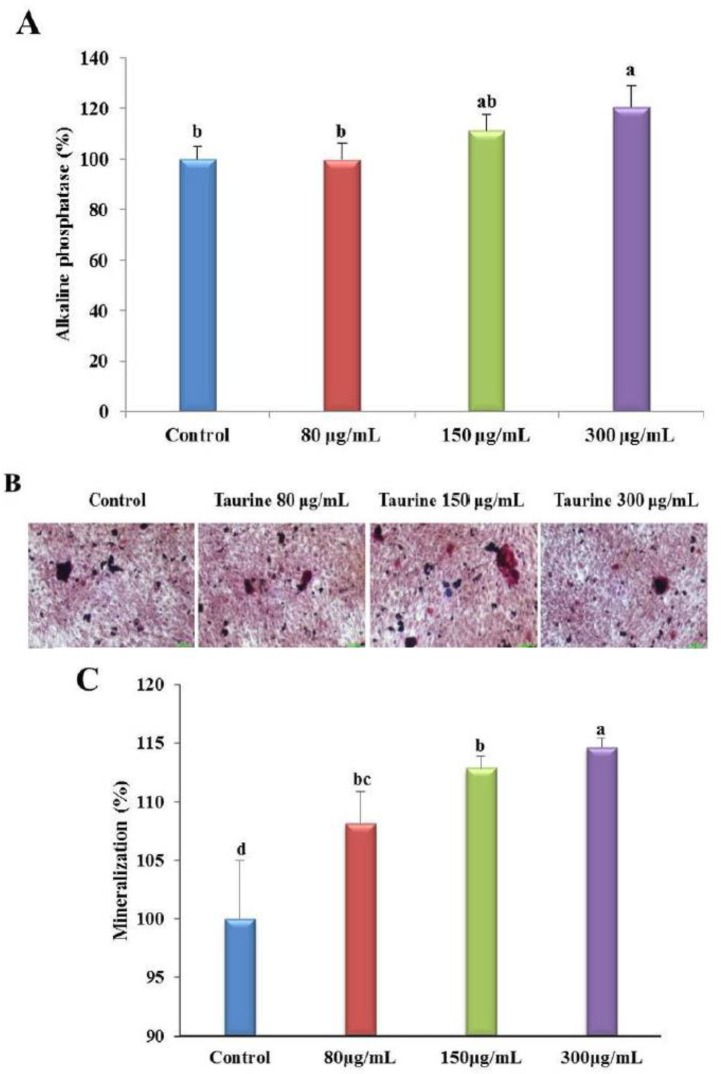
MG-63 cells were evaluated for cell viability, ALP activity, and mineralization rate for each concentration. (**A**) The level of ALP activation when control was set to 100%; (**B**) Details of mineralization analysis by using Alizarin red S staining; (**C**) The value of mineralization when control was set to 100%. Results are expressed as the mean and standard deviation (SD). Values not sharing a common superscript (a, b, c, and d) differed significantly (Duncan’s multiple range test)

The ALP activity of taurine-treated MG-63 cells was determined during the osteoblast differentiation stage. Taurine-treated MG-63 cells showed significant increase in ALP activity in a concentration-dependent manner (Fig. 1A). The ALP activity increased after treatment with 80 μg/mL taurine, but not significantly. The cellular ALP activity increased by 111% and 112% after treatment with 150 and 300 μg/mL taurine compared to that in the control cells, respectively. This suggests that taurine stimulates osteoblast differentiation.

The effect of taurine on the mineralization of MG-63 cells was investigated by using Alizarin red S staining and calcium deposition analysis. The taurine-treated MG-63 cells showed clear red colored bone nodules, unlike that in the control cells (Fig. 1B). Further, mineralization increased dose-dependently after treatment with taurine. These results suggest that taurine increases bone nodule formation in MG-63 cells (Fig. 1C). Compared to that in the controls, the degree of bone mineralization was increased by 108%, 112%, and 114% in cells treated with 80, 150, and 300 μg/mL of taurine (Fig. 1C). Therefore, taurine was found to effectively promote bone mineralization in MG-63 cells.

### Several signaling pathways and the expression of some proteins were increased by taurine in MG-63 cells

**Fig. 2:**
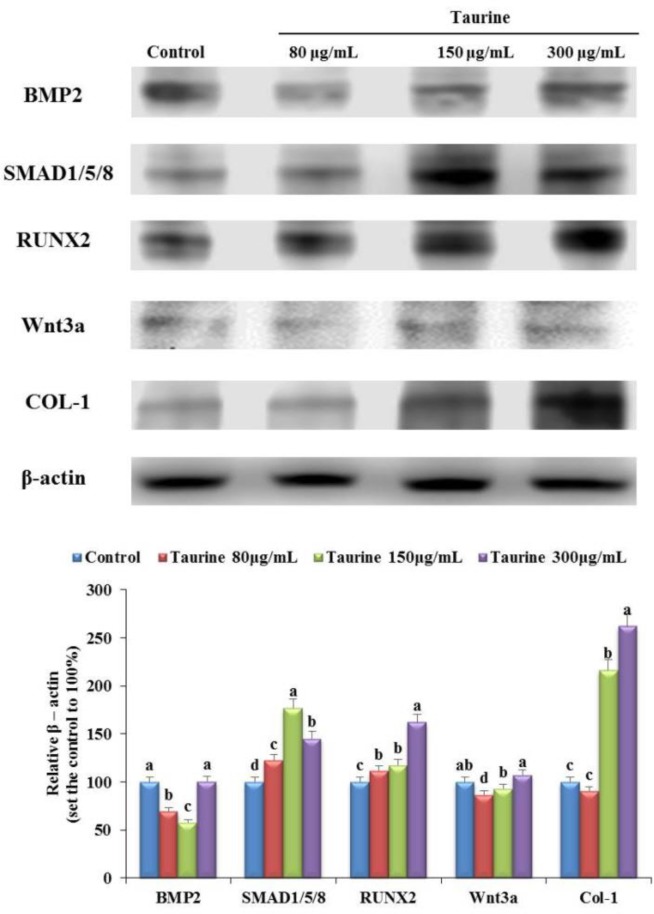
The expression of protein levels of molecules involved in bone metabolism in control, and 80 μg/mL, 150 μg/mL, and 300 μg/mL taurine-treated groups. The protein expression of BMP-2, small mothers against decapentaplegic (SMAD)1/5/8, RUNX2, Wnt3a, and COL-1 in control and treatment groups. Results are expressed as the mean and standard deviation (SD). Values not sharing a common superscript (a, b, c, and d) differed significantly (Duncan’s multiple range test)

Western blot revealed changes in the expression of BMP-2, SMAD1/5/8, RUNX2, Wnt3a, and COL-1. Treatment with 80 μg/mL taurine significantly increased the expression of SMAD1/5/8 and RUNX2 by 122% and 111%, respectively, compared with that in the control (Fig. 2). However, the expression of Wnt3a and COL-1 did not change, and that of BMP-2 decreased. Treatment with 150 μg/mL of taurine increased SAMD1/5/8, RUNX2, and COL-1 expression by 177%, 118%, and 216%, respectively, compared with that in the control, but decreased BMP-2 and did not affect Wnt3a expression. Treatment with 300 μg/mL taurine increased SAMD1/5/8, RUNX2, and COL-1 expression by 145%, 162%, and 261%, respectively, compared with that in the control, whereas decreased that of BMP-2 and did not affect Wnt3a expression (Fig. 2). These results suggest that taurine stimulates osteogenesis signaling pathways through osteoblast markers.

### Effects of taurine on the phosphorylation of Akt, extracellular signal-regulated kinase (ERK), c-Jun N-terminal kinase (JNK), and p38 MAPKs in MG-63 cells

**Fig. 3:**
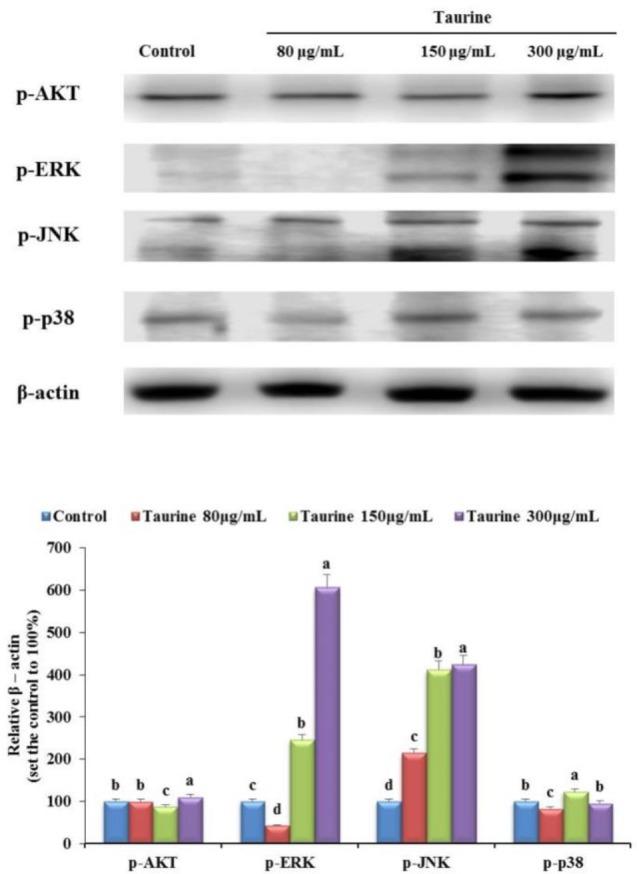
The expression of protein levels of molecules involved in bone metabolism in control, and 80 μg/mL, 150 μg/mL, and 300 μg/mL taurine-treated groups. The level of phosphorylated Akt, ERK, JNK, and p38 in control and treatment groups. Results are expressed as the mean and standard deviation (SD). Values not sharing a common superscript (a, b, c, and d) differed significantly (Duncan’s multiple range test)

We investigated whether phosphorylated Akt, ERK, JNK, and p38 proteins affected osteogenesis. Treatment with 80 μg/mL taurine changed the expression of all proteins except of *p*-JNK (Fig. 3). However, the activity of *p*-ERK and *p-*JNK was higher in the 150 and 300 μg/mL taurine-treated groups than in the control. In the 300 μg/mL taurine-treated group, the activity of *p*-Akt, *p*-ERK, and *p*-JNK significantly increased by 110%, 600% and 420% compared to that in the control, respectively (Fig. 3). However, no significant difference in *p*-p38 protein expression was noted. These data showed that taurine stimulates the phosphorylation of ERK and JNK. Thus, phosphorylation of ERK and JNK by taurine induces the proliferation and differentiation of osteoblastic MG-63 cells through the MAPK signal pathway.

### Treatment with 1% taurine increases several signaling pathways and the expression of some proteins in OVX rats

**Fig. 4:**
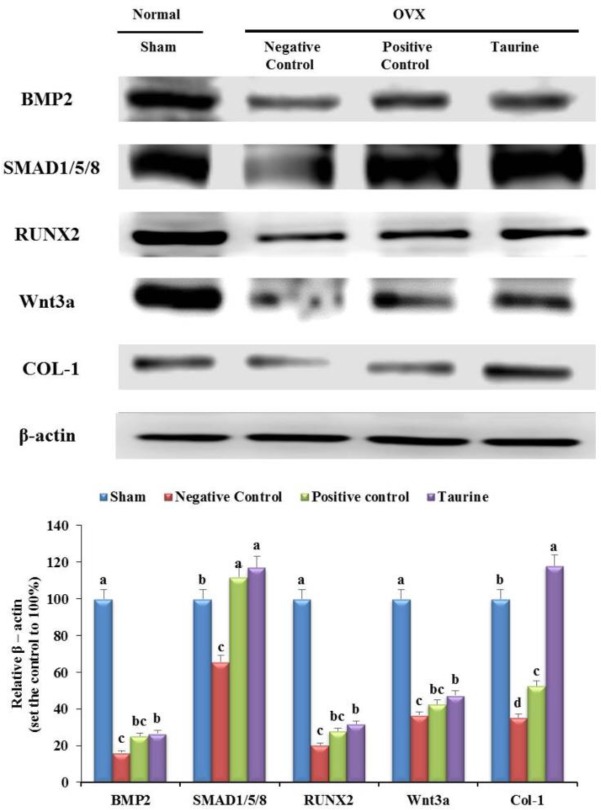
The protein expression levels of molecules involved in bone metabolism in the sham, negative control, positive control, and taurine groups. The protein expression of BMP-2, SMAD1/5/8, RUNX2, Wnt3a, and COL-1 in the sham, negative control, positive control, and taurine groups. The expression level of phosphorylated Akt, ERK, JNK, and p38 in the sham, negative control, positive control, and taurine groups. Results are expressed as the mean and standard deviation (SD). Values not sharing a common superscript (a, b, c, and d) differed significantly (Duncan’s multiple range test)

Western blot analysis performed on proteins extracted from the tibia and on osteoblast-related proteins showed that taurine significantly increased the expression of BMP-2, SMAD1/5/8, RUNX2, Wnt3a, and COL-1 (Fig. 4). Their expression tended to decrease by 16%, 66%, 20%, 37%, and 35%, respectively, in the negative control, whereas increased by 10%, 51%, 12%, 10%, and 83%, respectively, after treatment with taurine (Fig. 4). Further, their expression increased by 9%, 46%, 8%, 6%, and 18%, respectively, in the positive control, group compared with that in the negative control group (Fig. 4). Thus, taurine treatment significantly affected the expression of the five osteogenic factors compared with that in the negative control group (Fig. 4). This suggests that taurine significantly increases the expression of specific genes to prevent bone damage in OVX rats and promote osteoclast differentiation.

### Effects of 1% taurine on the phosphorylation of Akt, ERK, JNK, and p38 MAPKs in OVX rats

**Fig. 5:**
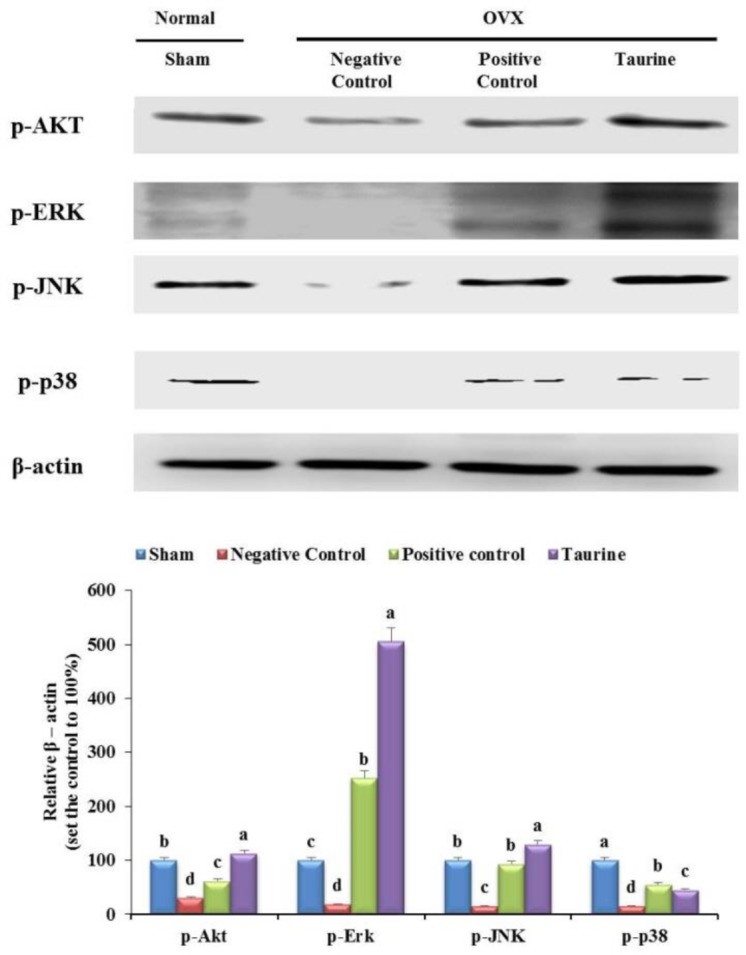
The protein expression levels of molecules involved in bone metabolism in the sham, negative control, positive control, and taurine groups. The expression level of phosphorylated Akt, ERK, JNK, and p38 in the sham, negative control, positive control, and taurine groups. Results are expressed as the mean and standard deviation (SD). Values not sharing a common superscript (a, b, c, and d) differed significantly (Duncan’s multiple range test)

The levels of Akt, ERK, JNK, and p38, which are activated by phosphorylation in association with osteoblast differentiation and proliferation, were measured. Their gene expression levels, measured using western blot were significantly increased in the taurine group (83%, 488%, 114%, and 30%, respectively) than in the negative control group (30%, 18%, 16%, and 15%, respectively) (Fig. 5). Further, their levels were increased by 32%, 235%, 77%, and 40%, respectively, in the positive control group than in the negative control group (Fig. 5). This result suggests that taurine stimulates osteoblast differentiation and proliferation by stimulating *p*-Akt, *p-*ERK, *p-*JNK, and *p*-p38 proteins in OVX rats.

## Discussion

Taurine, a water-soluble two-carbon atom amino acid, can be a promising therapeutic tool to reduce the risk of bone metabolic disorders such as osteoporosis. In this study, we investigated whether taurine increased osteoblast differentiation in MG-63 cells and bone metabolism protein expression in OVX rats. Further, to our knowledge, this is the first study on the regulatory effects of taurine on the osteogenic-related marker BMP-2, Wnt3a, SMAD1/5/8, RUNX2, and COL-1 via activation of the Akt and MAPK pathways.

ALP is an important indicator of bone mineralization; it is an early biomarker of bone metabolic disease and osteopenia and is expressed in all osteoblasts (22). As previously reported, the taurine transporter maintains a constant level of taurine in the bone tissue, is also expressed in osteoblasts, performs important functions of the bones, and promotes osteoblast differentiation. (8). Further, taurine is very safe in terms of tissue toxicity and is used as a supplement to drugs, baby milk, or health foods by pharmaceutical companies (19). In this study, taurine treatment of MG-63 cells resulted in a significant increase in ALP activity at 150 and 300 μg/mL compared with that in the control (Fig. 1). In addition, mineralization analysis by using alizarin red S staining of the taurine treatment group showed a significant and concentration-dependent increase in staining compared with that noted in the control (Fig. 1C). OVX rat model reduced the expression of osteoblast-forming genes such as BMP-2, Wnt3a, SMAD5, RUNX2, and COL-1, as previously reported (23–26). During osteoblast differentiation, BMP-2 stimulation is highly specific for osteoblastic transcription factors such as RUNX2, and these transcription factors play a pivotal role in the regulation of molecules such as ALP and osteocalcin during osteoblast differentiation (27). Our results suggest that taurine activates BMP-2, Wnt3a, SMAD1/5/8, and COL-1, through ALP both *in vitro* and *in vivo* and thus successfully improves bone formation and differentiation.

In addition, the association of Wnt3a with the PI3k-Akt signal has been reported, and the Akt signaling pathway has been shown to be associated with calcification through RUNX2 activation and expression. Previous studies have shown that the MAPK/ERK pathway is essential for controlling osteoblast differentiation and proliferation (28, 29). Various signaling pathways, including SMADs, Akt, and MAPK, have been reported to be activated upstream of RUNX2 expression during bone formation (30–31), with COL-1 being the downstream targets (29). Previous studies showed that, even at a concentration of 0.1 mM, taurine stimulated ERK 1/2 phosphorylation within 1 min, and the stimulatory action of taurine was blocked by an ERK 1/2 inhibitor (19). Important observation presented previously show that specific ERK 1/2 antagonist significantly attenuate magnesium levels of taurine in HOB cells, which increases magnesium levels through activation of ERK 1/2 in HOB cell (18). Further, another study found that taurine increased the nuclear localization of Cbfa1, and that this effect was mediated via the ERK signaling pathway (17). In our study, the expression of COL-1 was significantly increased after treatment with taurine in MG-63 cells and OVX rats (Fig. 2, 4). Our results showed an increase in the expression level of osteoblast markers (BMP-2, SMADs, Wnt3a, RUNX2, COL-1, *p*-MAPK, and *p*-Akt) in response to taurine treatment in differentiated MG-63 cells and OVX rats. Thus, our study results strongly suggest that taurine regulates osteoblast differentiation through Wnt3ainducible *p*-Akt/RUNX2 and BMP-mediated SMADs/MAPK/RUNX2 interactions.

## Conclusion

To our knowledge, this is the first report showing that treatment with taurine increased RUNX2 expression by activating Akt and MAPK through an increase in BMD, BMP-2, and Wnt3a levels. In addition, taurine increased the expression levels of BMP-2, Wnt3a, SMAD1/5/8, RUNX2, and COL-1 in the *in vivo* osteoporosis model and stimulated the *p*-Akt, *p*-p38, *p*-JNK, and *p*-ERK signaling pathways. These changes play an important role in improving the overall bone strength. Our study provides evidence that taurine can reduce the risk of bone metabolic disorders.

## Ethical considerations

Ethical issues (Including plagiarism, informed consent, misconduct, data fabrication and/or falsification, double publication and/or submission, redundancy, etc.) have been completely observed by the authors.
